# Recent Findings Unravel Genes and Genetic Factors Underlying *Leptosphaeria maculans* Resistance in *Brassica napus* and Its Relatives

**DOI:** 10.3390/ijms22010313

**Published:** 2020-12-30

**Authors:** Aldrin Y. Cantila, Nur Shuhadah Mohd Saad, Junrey C. Amas, David Edwards, Jacqueline Batley

**Affiliations:** School of Biological Sciences, University of Western Australia, Perth, WA 6009, Australia; aldrin.cantila@research.uwa.edu.au (A.Y.C.); nur.mohdsaad@research.uwa.edu.au (N.S.M.S.); junrey.amas@research.uwa.edu.au (J.C.A.); dave.edwards@uwa.edu.au (D.E.)

**Keywords:** *Brassica napus*, blackleg, resistance genes

## Abstract

Among the *Brassica* oilseeds, canola (*Brassica napus*) is the most economically significant globally. However, its production can be limited by blackleg disease, caused by the fungal pathogen *Lepstosphaeria maculans*. The deployment of resistance genes has been implemented as one of the key strategies to manage the disease. Genetic resistance against blackleg comes in two forms: qualitative resistance, controlled by a single, major resistance gene (*R* gene), and quantitative resistance (QR), controlled by numerous, small effect loci. *R*-gene-mediated blackleg resistance has been extensively studied, wherein several genomic regions harbouring *R* genes against *L. maculans* have been identified and three of these genes were cloned. These studies advance our understanding of the mechanism of *R* gene and pathogen avirulence (*Avr*) gene interaction. Notably, these studies revealed a more complex interaction than originally thought. Advances in genomics help unravel these complexities, providing insights into the genes and genetic factors towards improving blackleg resistance. Here, we aim to discuss the existing *R*-gene-mediated resistance, make a summary of candidate *R* genes against the disease, and emphasise the role of players involved in the pathogenicity and resistance. The comprehensive result will allow breeders to improve resistance to *L. maculans,* thereby increasing yield.

## 1. Introduction

The Brassicaceae family consists of diverse members, comprised of 372 genera and 4060 species [1]. The members include, but are not limited to, domesticated and wild root vegetables like turnip (*Brassica rapa* ssp. *rapa*, ssp. *oleifera*), swede (*Brassica napus* var. *napobrassica*), kohlrabi (*Brassica oleracea* var. *gongylodes*), radish (*Raphanus sativus*), and leafy vegetables (*B. rapa* ssp. *chinensis*, *B. oleracea* var. *viridis*, var. *acephala*, *Eruca sativa*, *Diplotaxis tenuifolia*) like cabbages (*B. oleracea* var. *capitata*, *Brassica fruticulosa*, *Coincya monensis*), broccoli (*B. oleracea* var. *italica*), cauliflower (*B. oleracea* var. *botrytis*), Brussel sprouts (*B. oleracea* var. *gemmifera*), mustards (*Brassica juncea*, *Brassica nigra*, *Brassica carinata*, *Brassica elongata*, *Hirschfeldia incana*, *Sinapis arvensis*, *Sinapis alba*), oilseed crops (*B. napus*, *B. rapa*, *B. juncea, Camelina sativa*), and a model plant (*Arabidopsis thaliana*). Interspecific hybridisations between diploid *B. rapa* (AA, 2n = 20), *B. nigra* (BB, 2n = 16), and *B. oleracea* (CC, 2n = 18) resulted in allotetraploid *B. juncea* (AABB, 2n = 4x = 36), *B. napus* (AACC, 2n = 4x = 38), and *B. carinata* (BBCC, 2n = 4x = 34), as shown in the triangle of U [2]. These Brassicaceae plant species have gained economic importance as condiments, dyes, medicinal uses, scientific models, ornamentals, vegetables, and the profitable canola oilseed [3,4,5,6,7,8]. The International Food Standards identified canola oil as those with low-erucic acid varieties from polyploid *B. napus* and *B. juncea* or diploid *B. rapa* [9].

Oilseed *Brassicas* are ranked second behind soybean in terms of worldwide production, with 75 million tonnes with an estimated value of 62.23 billion USD, cultivated over 38 million hectares in 63 countries in 2018 [10]. The top five producing countries, Canada, China, India, France and Australia, share 68% and 72% of the total production and cultivation, respectively (Table 1) [10]. Canola oil is recommended by health experts due to the low levels of saturated fat and high levels of omega-3 and -6 [11,12]. The oil can also be used for the production of margarine and as an additive for biodiesel, feedstock, fertilizer, adhesives, plastics and lubricants. The world export rate for canola oil and derived products is expected to rise from 20% to 40% in the coming years [13].

Blackleg disease, caused by the fungal pathogen *Leptosphaeria maculans*, is considered as one the main constraints to *B. napus* production [14,15,16]. The pathogen inoculum is disseminated through air and rain splashes [17], and may remain in infected crop residues for many years through the production of fruiting bodies (pycnidia and pseudothecia) [18,19]. *L. maculans* is a highly adapted fungal pathogen, capable of infecting all parts of the canola plant. Initially, spores enter into leaf openings or wounds, where they initiate a biotrophic mode of infection and eventually transition to a necrotrophic lifestyle as they find their way into the stem, leading to stem canker development. This stem colonization disrupts the nutrient flow and affects metabolic processes, ultimately killing the plant [20,21,22,23,24,25,26,27].

The first reported blackleg outbreak in *Brassica* was documented in *B. oleracea* [28]. Significant losses in *B. napus* were later reported in 1961 and 1972 in Canada and Australia, respectively [29,30,31]. An average of 10 to 20% annual yield losses is associated with this disease across canola-growing regions [19,32,33,34,35]. In uncontrolled conditions, losses range between 30% and 50% [36], with severe loss correlated to early seedling stage infection, particularly in the four- to five-leaf stage [36,37]. One of the most damaging incidences was documented in the Eyre Peninsula of South Australia following the breakdown of resistance of the cultivar Surpass 400 in 2003. This outbreak resulted in 90% production loss, equating to approximately 7.3 million USD [38,39].

Plants protect themselves by providing non-specific barriers in physical forms, such as a rigid cell wall [40,41], and chemical systems, such as producing proteins, sugars, lipoglycans and endotoxins [42,43]. When these barriers are overcome by pathogens, plants initiate a two-layered immunity response. The first layer involves detection of the pathogen-associated molecular patterns (PAMP) by the surface-localized receptors; this phase is termed PAMP-triggered immunity or PTI. However, PTI usually only leads to a mild defense response [44,45], which can be overridden by some pathogen races. As a counter response, the second layer of inducible response, called effector-triggered immunity (ETI), is initiated. This response relies on the interaction of a plant resistance gene (*R* gene), encoding recognition receptors, with race-specific pathogen effectors, encoded by avirulence (*Avr*) genes. This interaction usually leads to a hypersensitive defense response, which is usually manifested in rapid cell death, thereby limiting further pathogen growth, and phenotypically observed as complete resistance [45,46,47].

Based on conserved motifs, domains and features, *R* genes may be grouped into different classes collectively known as resistance gene analogs (RGAs). Three broad classes of RGAs are known: nucleotide-binding site-leucine rich repeats (NLRs), receptor-like protein kinases (RLKs), and receptor-like proteins (RLPs) [44,48,49]. Among them, NLRs are the largest class of RGAs predominately involved in plant disease resistance [50,51,52], whilst surface-localised RLKs and membrane-associated RLPs are pattern recognition receptors and integral components of the first line of defense [49,53,54], and have also been known to be involved in growth and development processes in plants [55].

**Figure 1:**
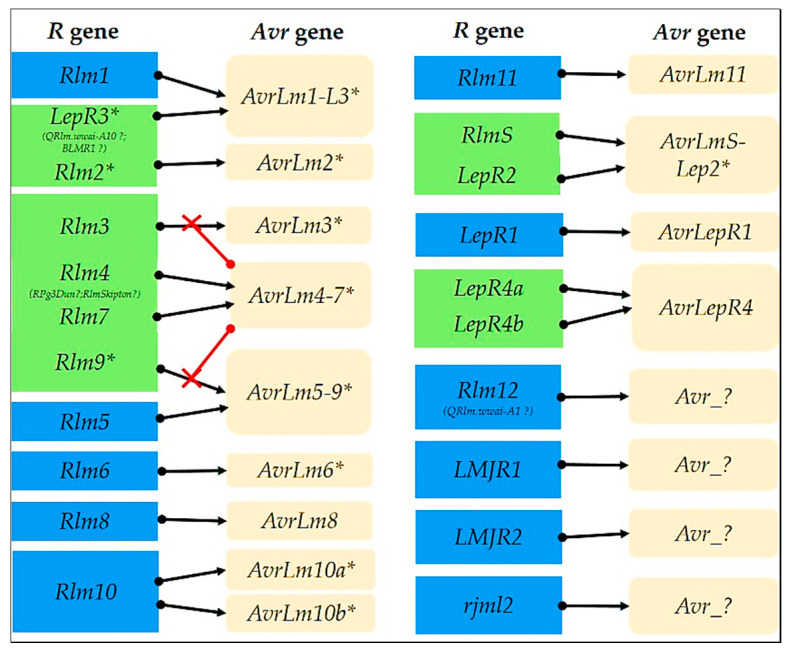
The complex interaction between resistance (*R*) genes and counterpart avirulence (*Avr*) genes mediating blackleg resistance in canola. *R* genes located in the same block (green) are allelic or suspected to be allelic forms. *Avr* genes that mask other interactions are indicated by an “x” sign. Genes (*R* and *Avr*) with an asterisk represent cloned genes. *Avr* genes with a question mark (?) are hypothetical genes that have not been isolated/discovered to date.

**Figure 2:**
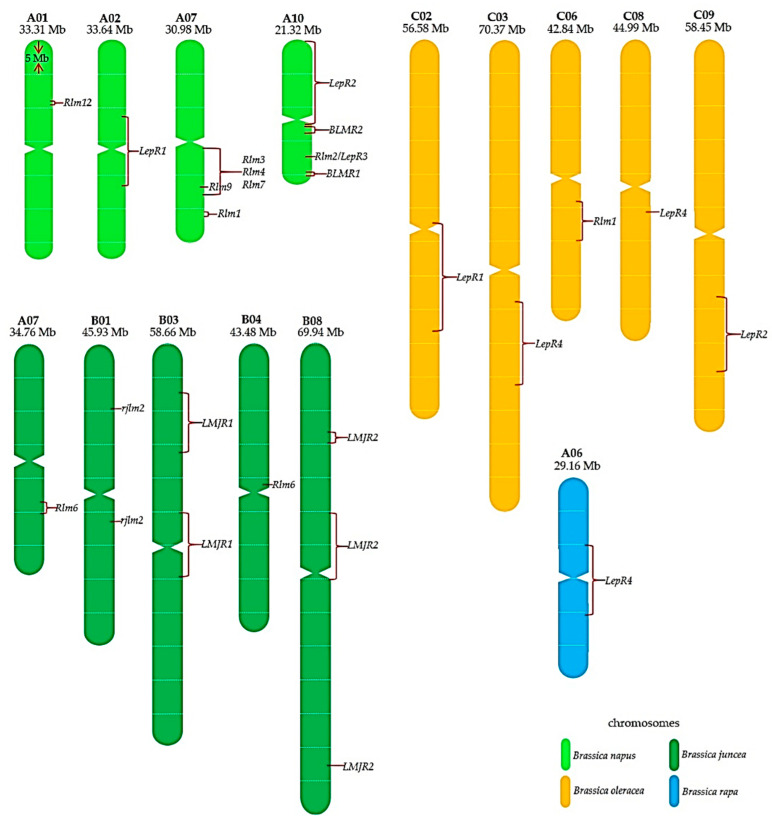
Current physical location of the known blackleg *R* genes based on quantitative trail loci (QTL) and candidate gene positions. Mb = million base pairs; *B. napus* pangenome [69,70,71], *B. oleracea* pangenome [72,73], *B. juncea* genome v. 1.5 [74] and *B. rapa* genome v. 3.0 [75].

There are two mechanisms controlling blackleg resistance. Qualitative resistance is generally controlled by a single major gene and is race-specific seedling resistance, active from the cotyledon through to the adult plant [56,57,58,59], while quantitative resistance is governed by multiple minor genes and is a partial resistance that is expressed in the later stages at leaf petioles and stem tissues [32,60]. To date, at least 16 *R* genes against blackleg have been genetically mapped in *B. napus* and other *Brassica* species (Figure 1 and Figure 2, Table 2). Of these, three have been cloned, and some of the 13 other genes are suspected to be identical or allelic forms due the different populations and markers used in their mapping (Figure 2). While *R* genes have been known to effect complete resistance, some *R* genes (*Rlm1, Rlm3, Rlm6, Rlm7, LepR1*, and *LepR3*) have been reported to break down and lose effectiveness in the field [38,61,62,63,64,65,66]. Currently, the deployment of *R* genes by crop rotation in canola cultivars is an integral approach to sustainably manage canola cultivation against blackleg infection and resistance breakdown [35,67,68].

This review focuses on the gene for gene mechanism of blackleg resistance, *R* gene content in canola and its relatives, candidate blackleg *R* genes, genetic factors in *L. maculans* pathogenicity and resistance, and future work that can advance knowledge towards a more resistant canola crop.

## 2. The Current Resistance Genes Go Beyond Simple Allelism

The flax–rust interaction provided some of the first evidence of a gene for gene interaction between plants and pathogens, whereby resistance is conferred by the highly specific recognition between the plant *R* genes and the pathogen’s *Avr* genes [93]. This molecular interaction initiates a cascade of signalling pathways, resulting in a hypersensitive response in the plant, which restricts further pathogen growth [94] and in some cases leads to systemic acquired resistance (SAR) [95]. Whilst the flax–rust interaction laid the foundation for understanding the basic mechanisms of *R*-gene-mediated resistance in plants, recent advances indicate a rather complex interaction in several crop-pathosystems, which goes beyond the simple gene-for-gene recognition. Such a case has been documented in the *Brassica- L. maculans* interaction, where several *R* genes have been found to interact with the same *Avr* genes in the pathogen, and in some instances, some *R* gene–*Avr* pairs mask the resistance response in other interactions. On the side of the host, several genes are suspected to be allelic forms of other genes, adding another layer of complexity for understanding the *Brassica–L. maculans* interaction.

*Rlm2* is a natural allele for resistance in *B. napus,* while *LepR3* is an introgressed gene from *B. rapa* subsp. *sylvestris*; however, subsequent investigations proved they are variants of the same gene [96,97]. *Rlm2* and *LepR3* are located on chromosome A10 (14,404,296 to 14,408,251 bp of *B. napus* Darmor-bzh genome v4.1) [98] and encode an extracellular leucine-rich receptor (RLP), whose structure was found to be similar to the widely known *Cf-a* protein in tomato [99,100]. Further functional analysis of both of these genes found that their resistance expression is mediated by associating with the helper proteins *SOBIR1* (Suppressor of BIR1) and *BAK1* (*BRI1-Associated Kinase-1*) proteins [97,100,101].

Whilst *Rlm2* and *LepR3* are allelic, they each recognise different *L. maculans Avr* genes; *Rlm2* interacts with *AvrLm2* while *LepR3* interacts with *AvrLm1*. The interaction of *LepR3* and *Rlm1* with the same *Avr* gene (*AvrLm1*) originating from different *Brassica* species [96,97] provides evidence of a two-for-one gene interaction for blackleg resistance (*Rlm1* and *LepR3-AvrLm1*), deviating from the earliest classical gene-for-gene interaction [93]. Recently, *LepR2* and *RlmS,* reported as independent genes, were found to interact with the same *Avr* gene, *AvrLmS-Lep2* [102]. However, since *LepR2* and *RlmS* are from *B. rapa* subsp. *sylvestris,* they could be the same gene or allelic variants. Cloning of *LepR2*, *RlmS*, and *Rlm1* will help explain why two genes recognise the same *Avr* gene.

*Rlm5* and *Rlm9* also recognise the same *Avr* gene (*AvrLm5-9*) [103]. *Rlm5* is a *B juncea R* gene that resides in a region homologous to chromosome A10 of *B. napus* [104,105]. *Rlm9* is on chromosome A07 (15.9 Mb in *B. napus* Darmor-bzh genome v4.1) and encodes a wall-associated kinase-like protein RLK [106]. However, as with the case of *LepR3* and *Rlm1,* it is unclear if *Rlm9* and *Rlm5* are allelic variants or independent genes [103]. Only when *Rlm5* is cloned can the relationship of these two genes be further dissected, which will explain why they share the same *Avr* gene.

In another interaction*,* genes *Rlm4* and *Rlm7* both recognise the same effector [107]. *Rlm4* and *Rlm7,* along with *Rlm3* and *Rlm9*, form a tightly linked cluster on chromosome A07 of *B. napus*, and may be alleles of the same *R* locus [108]. This hypothesis has a valid precedence as shown in the case of *Rlm2* and *LepR3,* which were found to be allelic [109].

A further gene, a *B. rapa* subsp. *sylvestris R* gene, *LepR4* is mapped on chromosome A06 (9,873,739 to 10,977,390 bp) in *B. rapa* v1.2 [110] but a recent finding in *B. oleracea* showed that *LepR4* candidate genes were detected on two different chromosomes, C03 and C08 [90,111]. Earlier, this gene was reported to have two alleles, *LepR4a* and *LepR4b*, each having different levels of resistance [86]. In *B. juncea*, another gene *Rlm6* has also been genetically mapped onto two different chromosomes, A07 and B04 [91]. Both *LepR4* and *Rlm6* are yet to be cloned. In *B. nigra*, the *R* gene *Rlm10* mapped on chromosome B04 interacts with two *Avr* genes, *AvrLm10a* and *AvrLm10b* [112,113], indicating a gene-for-two-gene interaction.

Other interactions seemed to follow the gene-for-gene, *R*-gene-to-*Avr*-gene interaction, and are relatively more straightforward to analyse compared with the previous examples. These include the interaction of *Rlm3* to *AvrLm3*, *Rlm8* to *AvrLm8*, *Rlm11* to *AvrLm11*, and *LepR1* to *AvrLepR1* [104,108,114,115]. However, as with most *R* genes, the specific genes controlling such resistance have yet to be closed. Hence, the identification of their sequences will likely contribute to how they should be effectively deployed for blackleg management.

Due to differences in the mapping population and the pathogen race compositions used, some blackleg *R* genes identified are thought to be redundant with other previously known *R* genes. Furthermore, their corresponding effectors remain to be verified. For example, *BLMR1* was thought to be redundant to *LepR3* [97] but an RNA sequencing analysis revealed a difference in the N-terminal leucine-rich repeat motifs [116] (Figure 1). *BLMR2, RPg3Dun*, *RlmSkipton*, and *QRlm.wwai-A10* are other genes that need confirmatory analysis [117,118,119] (Figure 1).

The simple gene-for-gene allelism provides a basic understanding in the *Brassica*-*L. maculans* interaction, however, complications exist for some of these genes, as some of them interact with the same *Avr* gene while several others are suspected to be allelic forms of the others. Furthermore, some of the interactions display an epistatic effect over the other interactions (Figure 1). These anomalies represent some of the challenges in studying the *Brassica*-*L. maculans* interaction, which need to be resolved to enhance current strategies for resistance deployment as a major component of blackleg management.

## 3. Exploring Resistance Genes in *Brassica napus* and Its Relatives

Despite natural resistance to *L. maculans* in the *B. napus* A-genome, there is a requirement to find novel sources of resistance for continuous improvement of the crop. One method for this is to utilise exotic germplasm via intergeneric/interspecific hybridisation breeding [120]. Several Brassicaceae species have been successfully hybridised/crossed with *B. napus* to improve resistance to blackleg, but information on the derived progenies is limited (Table 3). Only a few of these lines, containing *B. rapa* and *B. juncea* genes, have been successfully converted into commercial cultivars [104,121]. Other species including *A. thaliana, Brassica insularis, Brassica atlantica, Brassica macrocarpa, C. sativa, Diplotaxis muralis, Eruca pinnatifia, Erucastrum gallicum, Raphanus raphanistrum, S. alba, Sisymbrium loeselii*, and *Thlaspi arvense* have been found with proteins/compounds that may benefit *B. napus* against *L. maculans* [122,123,124,125,126,127,128,129,130,131,132,133,134]. Only 13 of these species have published information on their *R* gene content (Table 4). There are between 87–641 NLRs, 300–1,556 RLKs, and 56–272 RLPs in the genome assemblies [135,136,137,138,139,140,141,142,143] (Table 3).

It can be noted that RLKs are more abundant than other *R* genes. In the *B. napus* pangenome, across the 52 lines, there were 35,181 more RLK genes more than NLRs and 46,382 more RLK genes than RLPs, and in the 10 individuals in the *B. oleracea* pangenome, there were 316 more RLKs than NLRs and 709 more RLKs than RLPs [70,72]. The abundance of RLKs, over other *R*-genes type, could be due to their versatile roles in plants, as they are not only involved in defence but in other processes [156]. For example, RLKs are involved in growth and development such as cell proliferation and homeostasis, vascular differentiation, and steroid hormone perception [157,158,159]. RLKs interact with NLR/RLPs to initiate resistance [160,161,162,163,164,165,166,167] and their extracellular component suggests an ability to cope with the population of ligands from pathogens [168].

Most of the NLRs in the genome are involved in defence mechanisms [51] and some of the plant–pathogen interaction with effectors is indirect [169]. For example, a resistant tobacco with an NLR*-*N gene requires an *NRIP1* (NLR, specifically with TIR domain) before interacting with effector p50 of *Tobacco mosaic virus* [170]. A resistant *Arabidopsis* with *ZAR1* (NLR) requires *ZED1* pseudokinase (NLR) as a decoy, and thus *ZAR1*-mediated immunity is induced by interacting with type III *Avr HopZ1a* for resistance against *Pseudomonas syringae* [171]. NLRs perceive pathogen effector proteins in the cytoplasm, after which the plant initiates immunity through a hypersensitive response [172]. Of the 313 cloned *R* genes in plants, 191 are NLRs [169], with two NLRs in *Brassica*, *CRa* and *Crr1a*. *CRa* and *Crr1a* are resistant to isolates M85 and Ano-01 of *Plasmodiophora brassicae,* respectively, which causes clubroot disease in *B. rapa* [173,174].

RLPs are RLKs but without kinase domain, and usually an RLP gene would need other triggering genes to initiate resistance [54,175]. Aside from cloned RLP genes for resistance to *L. maculans*, other examples are *Cf-4* and *RLP23*. *Cf-4* perceives *Avr4* with the help of kinase-active *BAK1* to trigger an immunity response to *Cladosporium fulvum* in tomato [167]. *RLP23* requires *NEP*-like protein 20 and kinases (*SOBIR1* and *BAK1*) with the effector to signal an immune response to potato late blight and rot caused by *Phytophthora infestans* and *Sclerotinia sclerotiorum* [161,162].

Among the relatives of canola, B-genome-containing species (*B. nigra, B. carinata*, and *B. juncea*) are excellent sources of resistance to *L. maculans* [148,176,177]. Five *R* genes (*Rlm6*, *Rlm10*, *LMJR1, LMJR2,* and *rjlm2*) have been identified in the B-genome but only *Rlm6* is utilised in canola cultivars. Of the B genome species, *B. nigra* [178] and *B. juncea* [74] reference genomes have been published, while *B. carinata* genome assembly has yet to become available. Microsatellite markers indicated that resistance in *B. carinata* resides on chromosomes B01, B03, B06, and B07 in *B. napus-B. carinata* doubled haploid populations [145,146,147]. Nonetheless, studies can now rely on a pseudo-reference for *B. carinata* using its diploid ancestors: *B. nigra* and *B. oleracea* [179].

In other species, *A. thaliana* has been found to confer resistance to *L. maculans*; *RESISTANCE TO LEPTOSPHAERIA MACULANS (RLM) 1* or *AtRLM1A,* a 4.93 Kb gene on chromosome 1 (23,779,223 to 23,784,155 bp of *A. thaliana* Araport11), *AtRLM2* or *AtRLM1B,* a 5.59 Kb gene on chromosome 1 (23,711,420 to 23,717,006 bp of *A. thaliana* Araport11), and *AtRLM1A* [180,181,182]. *AtRLM1* and *AtRLM2* require camalexin production for resistance that causes lignification and the formation of vascular plugs as physical barriers [183,184]. *AtRLM3*, a 9.71 Kb gene on chromosome 4 9,557,175 to 9,566,887 bp of *A. thaliana* Araport11 [180], confers resistance not only to *L. maculans* but also to other diseases including *Botrytis cinerea*, *Alternaria brassicicola* and *Alternaria brassicae* [185]. *AtRLM3* has three *BREVIS RADIX* domains instead of leucine-rich repeats (LRR) that possibly regulates downstream defence signalling responses [186]. *AtRLM* gene homologs have been found in *Arabidopsis lyrata*, *B. rapa, C. sativa, Capsella rubella,* and *Eutrema salsugineum* based on annotation studies [181,186]. An *AtRLM1A*-like gene was identified in *C. rubella*, *AtRLM1B* and other *AtRLM1*-like genes in *A. lyrata*, *B. rapa* and *E. salsugineum;* and the *AtRLM3* gene is conserved in *A. lyrata* and *C. sativa* [181,186]. These species are potential sources to search for new resistance against *L. maculans*.

Other Brassicaceae relatives such as *C. monensis, S. arvenis, S. alba*, *D. muralis* and *Diplotaxis tenuifolia* have been found to have a resistance response against *L. maculans* in cotyledons and adult stages [122,123,132,133,154]. These species may contain vast numbers of disease resistance genes based on transcriptomic analysis [187,188,189]. The Brassicaceae, especially the wild relatives of *B. napus,* are indeed a potential source of novel *R* genes and alleles in improving resistance to *L. maculans* and for other diseases in the family. Their genome sequences provide an opportunity to search for orthologous allelic variants to the existing *R* genes for *L. maculans,* and a vast genetic resource that could considerably enrich *B. napus* in many years to come.

## 4. Genome Sequencing in *Brassica* Species Hastened the Identification of Resistance Genes

The availability of genome sequences marked a milestone in the identification of *R* genes and their cloning. *B. rapa* was the first *Brassica* species to have a genome sequence available [110]. Subsequently, the genome sequences of *B. napus, B. oleracea, B. juncea,* and *B. nigra* have become available [69,74,75,98,180,190,191], some of which have multiple genome assemblies. Recent genomic analysis has highlighted a significant gene presence absence variation in plant species, with disease resistance genes tending to demonstrate significant presence/absence variation [70,73,192,193,194]. This has led to the construction of pangenomes along with corresponding structural variation data including copy number and presence/absence variations for a wide range of crop species [195,196] including *Brassica* species [70,71,72,73,197].

The first two cloned genes for *L. maculans* resistance, *LepR3* and *Rlm2,* correspond to *BnaA10g20720D* and *Rlm9* to *BnaA07g20220D* in *B. napus* cv. Darmor bzh genome v4.1 [97,100,106], and the physical location has been updated in the *B. napus* pangenome (Figure 2). Most of the candidate genes for blackleg resistance encode RLKs followed by NLRs and RLPs, and a few encode TM-CCs, secreted peptides (SP) and enzymatic *R* genes (Table 2). These candidate genes can be useful a reference for researchers moving towards gene cloning and functional analyses. It is expected that the number of cloned *R* genes for blackleg resistance will increase in the near future.

## 5. Genetic Factors Involving the Pathogenicity and Resistance in *Leptosphaeria maculans*

Unlocking the genome of pathogens gives a better understanding of their pathogenicity, life-cycle, and evolution [198]. To date, 10 *L. maculans Avr* genes have been cloned (Figure 1). *AvrLm2*, *AvrLm3, AvrLm4-7*, *AvrLm5–9*, *AvrLm10a* and *AvrLm10b*, *AvrLm11,* and *AvrLmS-Lep2* encode cysteine-rich proteins [102,103,107,113,199,200,201,202,203], while *AvrLm1* contains only one cysteine residue [204].

All cloned and current candidate *Avr* genes reside in AT-rich sequences with degenerated transposable elements, where repeat-induced point mutation (RIP) often occurs [102,103,113,114,200,201,202,203,205]. As such, it was initially thought that RIP accounts for most of the virulence in *L. maculans* [109]. However, amino acid substitutions are the major cause of virulence, as occurs in *AvrLm2,* *AvrLm3, AvrLm4, AvrLm5*-*9* and gene deletions to *AvrLm1*, *AvrLm6*, *AvrLm10a* and *AvrLm10b*, *and AvrLm11* [103,109,113,114,200,201,206,207,208]. In *AvrLm7,* it is either RIP mutation or gene deletion causes virulence [109].

*AvrLm4-7* promotes *L. maculans* pathogenicity to susceptible *B. napus* and suppresses SA and ET signalling pathways, including abscisic acid (ABA) and hydrogen peroxide (H_2_O_2_) [209]. Similarly, *AvrLm1* suppresses SA and JA signalling pathways in transient gene expression of *A. thaliana* (Columbia-0 line) and targets phosphorylation of *B. napus* mitogen-activated protein kinase (*MAP_k*) 9 (*BnMAP_k9*) gene, which leads to an increase in cell death in *A. thaliana* [210]. As *AvrLm2* suppressed JA signalling, an MAP_k signal was induced; the mechanism could be similar to *AvrLm1* to *BnMAP_k9* gene but needs to further verification [211]. In a different study in *A. thaliana*–pathogen interaction, as the *AP2C1* gene (protein phosphatase gene) influenced *MAP_k4* and *MAP_k6* genes, the levels of JA and ET signalling genes were lowered, which subsequently compromised the plant immunity [212]. When MAP_k signalling genes were suppressed by *Xanthomonas* type III *Avr* genes (*XopE1, XopM, XopQ, AvrBs1* and *AvrXv4*), cell death occurs in *Nicotiana benthamiana* [213]. Another adenosine kinase has been found to be significant for proper fungal growth, hyphae development and virulence of *L. maculans* in *B. napus* [214]. *LmSNF1* (sucrose non-fermenting protein kinase 1 gene), *LmStuA* (TF gene), NEP1-like proteins, immunophilin gene family, isocitrate lyase, candidate secreted effector proteins, CAZymes, glycosyl hydrolase, cytokinin profiles, and carbohydrate with esterase domain containing genes play roles in *L. maculans* pathogenicity [215,216,217,218,219,220,221,222].

Generally, when *L. maculans* enters the plant, SA and JA-related genes are affected and act as initial defence compounds [84,209,216,217,218,223,224,225]. There are also genes that may contribute or act as basal defence, such as pattern recognition receptor *CERK1* (e.g., *chitin elicitor receptor kinase 1*), WRKY transcription factors (TF) (e.g., *WRKYs 33, 40* and *51*), glucosinolate-related genes (e.g., *cytochrome P450, SUPERROOT1,* and *nitrile-specifier protein 5*), and calcium-related biological functions (e.g., homologs of *CAM1*, *CAM5* and *CAM7; CYCLIC NUCLEOTIDE-GATED CALCIUM CHANNEL 3, 12* and *19; CALMODULIN-DOMAIN PROTEIN KINASE 5, 9*; *CALCIUM-DEPENDENT PROTEIN KINASE 6* and *28*; and *CALCINEURIN B-LIKE GENE 1*) [211,216].

When there is resistance, ABA is induced in plants harbouring *Rlm4, LepR3*, and *Rlm2* [116,209]. On the other hand, high expression of calcium-related signalling genes and TFs (basic leucine zipper (bZIP) and basic helix–loop–helix (bHLH)) aside from JA and ABA were found in plants containing *Rlm2* [211]. Calcium-dependent protein kinases have been reported to trigger signalling pathways for an immediate plant defence [226,227], while for TFs, bZIP acts as a precursor in plant immunity [228] and bHLH interacts with signalling plant defence receptors [229,230]. bHLH might have an important role in *Rlm2*-mediated defence, as it activates *SOBIR1* gene in *Gossypium barbadense* against *Verticillium* wilt [211,230]. Lastly, *LepR1*-mediated resistance was correlated with indole-derived phytoalexins [84], which may be a *Brassica*’s counterpart to camalexin that has been found to be effective against *L. maculans* [183,231].

## 6. Conclusions

*L. maculans* can adapt to the host over time in the field. Thus, canola breeders and scientists should use genomics and bioinformatics tools and platforms in *Brassica* research [232] to hasten the search for novel *R* genes for identification, cloning and deployment. The extensive applications of genomics, pangenomics, and superpangenomics to canola and its relatives [233] will result in genomic-driven breeding strategies. Additionally, applying these methodologies to the host will result in an *L. maculans*-informed canola breeding. We see transcriptomics uncover the *Brassica-L. maculans* interaction and reveal role players in the pathogenicity and resistance, which opens an opportunity for gene editing such as CRISPR technology by gene activation or inactivation [234,235,236,237]. Transcriptomics is also used to study the relatives of canola, which present a novel variation that may have natural and better resistance to the pathogen. Furthermore, physiological and other molecular mechanisms acting not only in the genes of canola but to other Brassicaceae species could also be explored for information which can be translated and useful in improving the crop [238]. The comprehensive information in this review allow breeders to integrate *Brassica* and *L. maculans*-sequencing-based information for developing a better and resistant *B. napus*.

## Figures and Tables

**Table 1:** The top 10 producing countries for canola with corresponding area harvested and yield in 2018 [10].

Country	Production (Tonnes, 10^6^)	Area Harvested (Ha, 10^6^)	Yield (Tonnes Per Ha)
1. Canada	20.34	9.12	2.23
2. China	13.28	6.55	2.03
3. India	8.43	6.70	1.26
4. France	4.95	1.62	3.06
5. Australia	3.89	3.17	1.23
6. Germany	3.67	1.22	3.00
7. Ukraine	2.75	1.04	2.65
8. Poland	2.20	0.84	2.64
9. USA	2.01	0.79	2.55
10. Russia	1.99	1.50	1.33

**Table 2:** List of candidate genes harbouring resistance to *Leptosphaeria maculans* with their reported resistance gene analog (RGA) function along with their closest gene ortholog having disease resistance/other function.

Gene (Position)	Candidate Genes	RGA Type	Gene Ortholog (TAIR10)	Molecular Function	References
*Rlm1* (A07 in Bna)	BnaA07g28760D	RLP	AT1G56140	LRR TM prot_k	[76]
	BnaA07g29310D	RLK	AT1G71390	RLP 11	[76]
	BnaA07g27720D	NLR	AT1G69160	BIG GRAIN LIKE 1 supressor	[76]
	BnaA07g28550D	-	AT1G33612	Receptor for the Plant Natriuretic Peptide	[76]
	BnaA07g28840D	RLK	AT1G70740	Prot_k superfam_prot	[77]
	BnaA07g27460D	RLK	AT1G68830	STN7 prot_k	[78]
*Rlm3, Rlm4 & Rlm7* (A07 in Bna)	BnaA07g20490D-		AT1G79090	Protein PAT1 homolog	[76]
	BnaA07g20910D	NLR	AT1G77610	UDP-galactose transporter 1	[76]
	BnaA07g17000D	NLR	AT1G12220	DRP RPS5/nucleotide binding	[79]
	BnaA07g17760D	RLK	AT1G56145	LRR TM prot_k	[79]
	BnaA07g18000D	RLK	AT3G58690	Prot_k superfam_prot	[79]
	BnaA07g18480D	RLK	AT3G59700	L-type lectin-domain containing receptor kinase V.5	[79]
	BnaA07g20630D	RLK	AT1G78290	SRK2C/ST_k	[79]
	BnaA07g18680D	TM-CC	AT3G60470	LRR TM prot_k	[79]
	BnaA07g18770D	TM-CC	AT3G60600	VAP 1-1/protein binding	[79]
	BnaA07g18880D	TM-CC	AT3G61050	NTMC2T4/lipid binding	[79]
	BnaA07g19680D	TM-CC	AT1G79830	GC5 (golgin candidate 5)/protein binding	[79]
	BnaA07g20240D	RLK	AT1G79640	Prot_k superfam_prot/ST_k tyrosine	[80]
*Rlm12* (A01 in Bna)	BnaA01g12900D	RLP	AT4G23100	Glutamate-cysteine ligase, chloroplastic	[81]
	BnaA01g12800D	RLP	AT4G22990	Major Facilitator Superfamily with SPX	[81]
	BnaA01g12940D	RLP	AT4G23240	Putative cysteine-rich RLP kinase 16	[81]
*LepR1* (A02 in Bna)	BnaA02g15610D	RLK	AT1G71870	Protein DETOXIFICATION 54/MATE efflux fam_prot	[70,76]
	BnaA02g15810D	RLK	AT1G72140	Protein NRT1/PTR FAMILY 5.12/proton-dependent oligopeptide transport (POT) fam_prot	[70,76]
	BnaA02g15820D	RLK	AT1G72150	Patellin-1/transporter	[70,76]
	BnaA02g15890D	RLK	AT1G72290.1 (CDS)	Cysteine protease inhibitor WSCP	[70,76]
	BnaA02g16700D	RLK	AT2G18910	Expressed protein/hydroxyproline-rich glycoprotein fam_prot	[70,76]
	BnaA02g16770D	RLK	AT1G74190	RLP 15	[70,76]
	BnaA02g16960D	NLR	AT1G30490.1 (CDS)	Homeobox-leucine zipper protein ATHB-9	[70,76]
	BnaA02g18160D	TM-CC	AT1G76570	Chlorophyll a-b binding protein 7, chloroplastic	[70,76]
	BnaA02g20380D	RLK	AT4G01440	WAT1-related protein	[70,76]
	BnaA02g20440D	RLK	AT4G01590	DNA-directed RNA polymerase III subunit	[70,76]
	BnaA02g20610D	RLK	AT4G02510	Translocase of chloroplast 159, chloroplastic/TM receptor	[70,76]
	BnaA02g21110D	RLK	AT5G19010	MAP kinase 16	[70,76]
	BnaA02g21890D	RLK	AT4G11010	Nucleoside diphosphate kinase/ATP binding	[70,76]
	BnaA02g22210D	RLK	AT5G43370	Probable inorganic phosphate transporter 1-2	[70,76]
	BnaA02g22280D	RLK	AT5G43710	Alpha-mannosidase/glycoside hydrolase family 47 protein	[70,76]
	BnaA02g22610D	NLR	AT5G40910	DRP (TNL class)	[70,76]
	BnaA02g23050D	TM-CC	AT5G42570	Intracellular protein transport	[82,83]
	BnaA02g24000D	NLR	AT5G45490	Probable DRP	[82,83]
	BnaA02g24440D	RLP	AT5G46330	LRR RLP kinase/TM ST_k	[82,83]
	BnaA02g24500D	NLR	AT5G46510	DRP (TNL class)	[82,83]
	BnaA02g24510D	NLR	AT5G46450	DRP (TNL class)	[82,83]
	BnaA02g24530D	NLR	AT5G46450	DRP (TNL class)	[82,83]
	BnaA02g24540D	NLR	AT5G46450	DRP (TNL class)	[82,83]
	BnaA02g24560D	NLR	AT5G46451	DRP (TNL class)	[82,83]
	BnaA02g25110D	NLR	AT5G47220	Ethylene responsive element binding factor 2	[84]
*LepR2* (A10 in Bna)	BnaA10g03460D	RLK	AT1G05300	Zinc transporter 5	[70,76]
	BnaA10g06440D	RLK	AT5G53070	Ribosomal protein L9/RNase H1	[70,76]
	BnaA10g07140D	RLK	AT3G15240	ST_k WNK (With No Lysine)-like protein	[70,76]
	BnaA10g09460D	NLR	AT5G55220	Trigger factor-like protein TIG, chloroplastic	[70,76]
	BnaA10g09870D	RLK	AT5G55670	RNA-binding (RRM/RBD/RNP motifs) fam_prot	[70,76]
	BnaA10g10000D	NLR	AT5G55910	ST_k D6PK	[70,76]
	BnaA10g12510D	RLK	AT5G59200	Putative pentatricopeptide repeat-containing protein, chloroplastic	[70,76]
	BnaA10g13610D	NLR	AT5G60000	TM protein	[70,76]
	BnaA10g14660D	RLK	AT5G20900	TIFY 3B/JAZ12 (JASMONATE-ZIM-DOMAIN PROTEIN 12)	[70,76]
	BnaA10g14840D	RLK	AT5G20670	Unknown protein	[70,76]
	BnaA10g06390D	RLK	AT5G53000	PP2A regulatory subunit TAP46	[70,76]
	BnaA10g07390D	RLK	AT5G52520	Proline--tRNA ligase, chloroplastic/mitochondrial	[70,76]
	BnaA10g07400D	RLK	AT5G52510	SCL8	[70,76]
	BnaA10g07410D	RLK	AT5G52510	SCL8	[70,76]
	BnaA10g07650D	RLK	AT5G51970	Sorbitol dehydrogenase	[70,76]
	BnaA10g09120D	RLK	AT5G54850	Unknown protein	[70,76]
	BnaA10g09500D	RLK	AT5G55280	Cell division protein FtsZ homolog 1, chloroplastic	[70,76]
	BnaA10g10380D	RLK	AT5G56220	P-loop containing nucleoside triphosphate hydrolases superfam_prot/nucleotide binding	[70,76]
	BnaA10g10430D	RLK	AT5G56210	WPP domain-interacting protein 2	[70,76]
	BnaA10g11120D	RLK	AT5G57110	Calcium-transporting ATPase	[70,76]
	BnaA10g11930D	RLK	AT5G58410	E3 ubiquitin-protein ligase listerin/zinc ion binding	[70,76]
	BnaA10g12560D	RLK	AT5G59610	Chaperone DnaJ-domain superfam_prot/DNAJ heat shock N-terminal domain-containing protein	[70,76]
	BnaA10g12830D	RLK	AT4G34110	Polyadenylate-binding/RNA binding/translation initiation factor	[70,76]
	BnaA10g12860D	RLK	AT5G59900	Putative pentatricopeptide repeat-containing protein	[70,76]
	BnaA10g12870D	RLK	AT5G22880	Histone H2B/DNA binding	[70,76]
	BnaA10g12880D	RLK	AT5G59950	RNA-binding fam_prot/RNA and export factor-binding protein	[70,76]
	BnaA10g12890D	RLK	AT5G59990	CCT motif fam_prot	[70,76]
	BnaA10g12900D	RLK	AT5G60020	Laccase-17	[70,76]
	BnaA10g12950D	RLK	AT5G60120	Target of early activation tagged (EAT) 2/TF	[70,76]
	BnaA10g14170D	RLK	AT5G22170	TM protein	[70,76]
	BnaA10g14640D	RLK	AT2G24080	F-box protein (DUF295)	[70,76]
	BnaA10g15480D	RLK	AT5G19690	Dolichyl-diphosphooligosaccharide-protein glycosyltransferase subunit STT3A	[70,76]
	BnaA10g18330D	RLK	AT5G16000	Protein NSP-INTERACTING KINASE 1	[70,76]
	BnaA10g19700D	RLK	AT5G13870	Xyloglucan endotransglucosylase/hydrolase	[70,76]
	BnaA10g20110D	RLK	AT5G13180	NAC domain-containing protein 83/TF	[70,76]
	BnaA10g23030D	RLK	AT5G08450	Zinc finger CCCH domain protein	[70,76]
	BnaA10g23040D	RLK	AT5G08440	Unknown protein	[70,76]
	BnaA10g26650D	RLK	AT5G03290	Isocitrate dehydrogenase (NAD) catalytic subunit 5, mitochondrial	[70,76]
*BLMR1* (A10 in Bna)	BnaA10g21910D	-	AT5G10360	40S ribosomal protein S6 (RPS6B)	[85]
	BnaA10g19660D	-	AT3G17620	Putative F-box domain protein	[85]
*BLMR2* (A10 in Bna)	BnaA10g11390D	-	AT5G57340	Ras guanine nucleotide exchange factor Q-like protein	[85]
	BnaA10g11500D	TM	AT5G57560	Xyloglucan endotransglucosylase/hydrolase	[85]
*LepR4* (A06 in Bra)	Bra018037	NLR	AT5G17680	DRP (TNL class)	[86]
	Bra018057	NLR	AT5G66900	DRP (CNL class)	[86]
	Bra018198	NLR	AT3G46710	DRP (CNL class)	[86]
	Bra019483	NLR	AT2G15530	RING/U-box superfam_prot	[86]
*Rlm1* (C06 in Bol)	Bo6g077080	NLR	AT3G60490	Ethylene-responsive TF ERF035 APETALA2	[87]
	Bo6g088090	RLK	AT1G73080	RLP kinase LRR-RLK, STKc	[87]
	Bo6g080150	RLK	AT1G80080	Protein TOO MANY MOUTHS_TMM LRR	[87]
	Bo6g093010	RLK	AT1G71830	Somatic embryogenesis receptor kinase 1 LRR-RLK, STKc	[87]
	Bo6g089160	NLR	AT1G72890	DRP (TIR-NBS class)	[87]
	Bo6g089290	NLR	AT1G72850	DRP (TIR-NBS class)	[88]
*LepR1* (C02 in Bol)	Bo2g093170	NLR	AT1G57850	TIR domain protein family	[88]
	Bo2g095430	LRR	AT1G22000	Putative F-box/LRR protein	[88]
	Bo2g095460	RLK	AT1G79620	LRR RLP kinase	[88]
	Bo2g103360	NLR	AT5G36930	DRP (TNL class)	[88]
	Bo2g103380	LRR	AT4G03220	Putative F-box/LRR protein	[88]
	Bo2g104830	LRR	AT3G47580	LRR RLP kinase	[88]
	Bo2g118150	RLK	AT1G56120	LRR TM prot_k	[88]
	Bo2g118200	RLK	AT1G56130	Probable LRR RLK ST_k	[88]
	Bo2g124490	NLR	AT1G63730	DRP (TNL class)	[88]
	Bo2g124590	RLK	AT5G44700	LRR RLK ST_k GSO2	[88]
	Bo2g125680	RLK	AT3G47570	Probable LRR RLK ST_k	[88]
	Bo2g125700	RLK	AT5G20480	LRR RLK ST_k	[88]
	Bo2g126850	NLR	AT5G45220	DRP (TNL class)	[88]
	Bo2g126860	NLR	AT2G17050	DRP (TNL class)	[88]
	Bo2g126870	NLR	AT5G45210	DRP (TNL class)	[88]
	Bo2g126880	NLR	AT5G17880	Disease resistance-like protein CSA1	[88]
	Bo2g126900	NLR	AT5G45220	DRP (TNL class)	[88]
	Bo2g126920	NLR	AT5G45230	DRP (TNL class)	[88]
	Bo2g126980	NLR	AT5G45240	DRP (TNL class)	[88]
	Bo2g127270	NLR	AT5G45490	Probable DRP	[88]
	Bo2g127290	NLR	AT5G45490	Probable DRP	[88]
	Bo2g127320	NLR	AT5G45510	Probable DRP	[88]
	Bo2g129990	RLK	AT5G46330	LRR RLP kinase	[88]
	Bo2g130040	NLR	AT5G46470	DRP RPS6	[88]
	Bo2g130050	LRR	AT5G40060	DRP (NLR class)	[88]
	Bo2g130080	NLR	AT5G46270	DRP (TNL class)	[88]
	Bo2g130090	NLR	AT5G46450	DRP (TNL class)	[88]
	Bo2g130100	NLR	AT5G46450	DRP (TNL class)	[88]
	Bo2g130110	NLR	AT4G08450	DRP (TNL class)	[88]
	Bo2g130150	NLR	AT4G08450	DRP (TNL class)	[88]
	Bo2g130180	NLR	AT5G46450	DRP (TNL class)	[88]
	Bo2g131530	NLR	AT4G16920	DRP (TNL class)	[88]
	Bo2g131540	NLR	AT5G46270	DRP (TNL class)	[88]
	Bo2g131590	NLR	AT5G46450	DRP (TNL class)	[88]
	Bo2g131610	NLR	AT5G46260	DRP (TNL class)	[88]
	Bo2g131620	NLR	AT5G40060	DRP (NLR class)	[88]
*LepR2* (C09 in Bol)	Bo9g111490	LRR	AT1G51370	F-box domain/LRR protein	[89]
	Bo9g111500	LRR	AT5G25850	Putative F-box domain/LRR protein	[89]
	Bo9g111510	LRR	AT5G53840	F-box domain/LRR protein	[89]
	Bo9g113780	RLK	AT5G53890	LRR RLP kinase	[89]
	Bo9g117290	RLK	AT5G54380	RLP kinase THESEUS 1	[89]
	Bo9g119130	RLK	AT5G55090	MAP kinase 15	[89]
	Bo9g120720	LRR	AT5G66330	LRR fam_prot	[89]
	Bo9g122300	RLK	AT5G56040	LRR RLP kinase	[89]
	Bo9g125930	LRR	AT3G56780	F-box domain/LRR protein	[89]
	Bo9g126120	LRR	AT5G56560	F-box domain/LRR protein	[89]
	Bo9g126140	LRR	AT5G56560	F-box domain/LRR protein	[89]
	Bo9g126150	RLK	AT5G56580	MAP kinase 6	[89]
	Bo9g135700	CC	AT2G42480	MATH & CC domain-containing protein	[89]
*LepR4* (C03 in Bol)	Bo3g099380	RLK	AT5G65240	LRR prot_k	[90]
	Bo3g102880	NLR	AT4G36150	DRP (TNL class)	[90]
	Bo3g103150	RLK	AT5G63710	LRR prot_k	[90]
	Bo3g107230	RLK	AT5G62710	LRR prot_k	[90]
	Bo3g107530	RLK	AT1G53510	MAP Pkinase 18	[90]
	Bo3g110840	RLK	AT3G47090	LRR prot_k	[90]
	Bo3g114980	RLK	AT3G47090	MAP Pkinase_Tyr, ST_k	[90]
	Bo3g130040	RLK	AT3G45640	MAP Pkinase	[90]
	Bo3g134690	RLK	AT3G47580	LRR protein Pkinase	[90]
*LepR4* (C08 in Bol)	Bo8g077170	RLK	AT1G53510	MAP Pkinase 18	[90]
	Bo8g077270	NLR	AT5G17680	DRP (TNL class)	[90]
	Bo8g077320	CC	AT3G48860	CC domain containing protein SCD2	[90]
Rlm6 (A07 in Bju)	BjuA027357	RLK	AT1G66830	Probable inactive LRR RLP kinase	[91]
	BjuA043308	RLK	AT1G67510	LRR prot_k fam_prot	[91]
*Rlm6* (B04 in Bju)	BjuB042709	RLK	AT1G10850	LRR prot_k fam_prot/ST_k	[91]
	BjuB042726	RLK	AT1G11130	LRR prot_k fam_prot/receptor signalling protein ST_k	[91]
*LMJR1* (B03 in Bju)	BjuB043144	RLK	AT1G61360	ST_k	[92]
	BjuB032936	RLK	AT1G29720	Probable LRR RLK ST_k	[92]
	BjuB043487	RLK	AT1G21209	Wall associated kinase 4	[92]
	BjuB043117	RLP	AT1G10520	DNA polymerase lambda	[92]
	BjuB047419	RLP	AT1G71400	RLP 12	[92]
*LMJR2* (B08 in Bju)	BjuB015599	RLK	AT5G59680	Probable LRR RLK ST_k	[92]
	BjuB041327	RLK	AT4G08850	MDIS1-interacting RLK 2	[92]
	BjuB040922	RLK	AT5G07620	Prot_k superfam_prot	[92]
	BjuB041021	RLK	AT5G10530	L-type lectin-domain containing receptor kinase IX.1	[92]
	BjuB019224	RLK	AT3G56050	Probable inactive RLP kinase	[92]
	BjuB045981	RLP	AT5G56810	Putative F-box domain/LRR protein	[92]
*rjlm2* (B01 in Bju)	BjuB026698	RLK	AT2G35620	LRR RLK ST_k FEI 2	[92]
	BjuB025797	RLK	AT5G38210	Prot_k fam_prot	[92]

TAIR10 = *Arabidopsis thaliana* genome reference with e-value hit ≤ 0.000003, Bna *= Brassica napus,* Bra *= Brassica rapa,* Bol *= Brassica oleracea,* Bju *= Brassica juncea*, RLP = receptor-like protein, RLK = receptor-like kinase, NLR = nucleotide binding site (NBS) leucine-rich repeat (LRR), TM-CC = transmembrane (TM) coiled-coil (CC), LRR = leucine-rich repeat, TNL = toll-interleukin-resistance (TIR)-NBS-LRR, CNL = CC-NBS-LRR, MAP = mitogen-activated protein, ST_k = serine/threonine protein kinase, SCL8 = scarecrow-like TF 8, DRP = disease resistance protein, prot_k = protein kinase, fam_prot =family protein, TF = transcription factor.

**Table 3:** Successful *Brassica napus* progenies hybridized/developed with selected Brassicaceae species having blackleg resistance in cotyledon stage.

Species	Types of Progenies	References
*Brassica carinata* and *Brassica rapa*	Double haploid (DH) lines	[86,115,144,145,146,147]
*Brassica juncea*	Recombinant and backcrossed (BC) lines	[148,149,150]
*Brassica nigra*	Hybrid and recombinant lines	[149,151,152]
*Brassica elongata*, *Brassica fruticulosa*, *Brassica souliei* and *Diplotaxis tenuifolia*	Hybrid	[130]
*Coincya monensis* and *Hirschfeldia incana*	Hybrid and BC lines	[130,132]
*Sinapsis arvensis*	Somatic hybrids and BC lines	[130,132,153,154]
*Brassica tournefortii*	Somatic hybrids	[155]

**Table 4:** List of Brassicaceae species containing resistance gene analogs like nucleotide-binding site leucine-rich repeat (NLR), receptor-like protein kinase (RLK), and receptor-like protein (RLP).

Species (Common Name)	NLR	RLK	RLP	Software Used	References
*Arabidopsis lyrata* (Lyre-leaved rock-cress)	243	514	73	RGAugury	[140]
	506	495	56	RGAugury	[138]
	198	-	-	HMM/MEME	[143]
	200	-	-	HMM/LRRfinder	[137]
*Arabidopsis thaliana* (Mouse-ear cress)	205	516	73	RGAugury	[140]
	410	517	75	RGAugury	[138]
	152	-	-	NLGenome Sweeper	[141]
	213	-	-	HMMER	[142]
	165	-	-	HMM/MEME	[143]
	167	-	-	HMM/LRRfinder	[137]
*Brassica juncea* (Indian mustard)	315	1085	191	RGAugury	[140]
	-	493	228	RGAugury	[91]
*Brassica napus* (Oilseed rape)	286 ^1^	989 ^1^	77 ^1^	RGAugury	[140]
	208 ^2^	680 ^2^	223 ^2^	RGAugury	[140]
	621 ^3^	1497 ^3^	273 ^3^	RGAugury	[140]
	566 ^4^	1517 ^4^	260 ^4^	RGAugury	[140]
	464	-	-	HMMER	[136]
	641	-	-	MEME/MAST	[135]
*B. napus* pangenome	16430	51611	5229	RGAugury	[70]
*Brassica nigra* (Black mustard)	372	776	176	RGAugury	[140]
	-	317	176	RGAugury	[91]
*Brassica oleracea* (Cabbage)	493	822	159	RGAugury	[72]
	438	796	155	RGAugury	[140]
	146	-	-	HMMER	[136]
	443	-	-	MEME/MAST	[135]
	157	-	-	HMMER	[142]
	408	-	-	HMMER	[139]
*B. oleracea* pangenome	616	932	223	RGAugury	[72]
*Brassica rapa* (Field mustard)	263	670	106	RGAugury	[140]
	488	747	118	RGAugury	[138]
	-	300	65	RGAugury	[91]
	202	-	-	HMMER	[136]
	249	-	-	MEME/MAST	[135]
	206	-	-	HMMER	[142]
	204	-	-	HMM/MEME	[143]
	201	-	-	HMM/LRRfinder	[137]
*Brassica macrocarpa*(‘Egadi‘ cabbage)	447	862	186	RGAugury	[72]
*Camelina sativa* (False flax)	504	1469	280	RGAugury	[140]
*Capsella rubella* (pink shepherd’s-purse)	180	539	87	RGAugury	[140]
	200	536	97	RGAugury	[138]
	127	-	-	HMM/MEME	[143]
*Eutrema salsugineum* (Saltwater cress)	165	509	77	RGAugury	[140]
	348	483	83	RGAugury	[138]
	88	-	-	HMM/MEME	[143]
	87	-	-	HMM/LRRfinder	[137]
*Raphanus raphanistrum* (Wild radish)	206	585	142	RGAugury	[140]
*Thlaspi arvense* (Field penny-cress)	183	474	120	RGAugury	[140]

^1^*Brassica napus* cv. Darmor v.8, ^2^ *Brassica napus* cv. Tapidor, ^3^ *Brassica napus* cv. Darmor v.4, ^4^ *Brassica napus* cv. ZS11.

## Data Availability

No new data were created or analyzed in this study. Data sharing is not applicable to this article.
